# Analysis of Photoluminescence Thermal Quenching: Guidance for the Design of Highly Effective p-type Doping of Nitrides

**DOI:** 10.1038/srep32033

**Published:** 2016-08-23

**Authors:** Zhiqiang Liu, Yang Huang, Xiaoyan Yi, Binglei Fu, Guodong Yuan, Junxi Wang, Jinmin Li, Yong Zhang

**Affiliations:** 1 Research and Development Center for Solid State Lighting, Institute of Semiconductors, Chinese Academy of Science,Beijing, 100086, China; 2 State Key Laboratory of Solid State Lighting, Beijing, 100086, China; 3 Department of Electrical and Computer Engineering, The University of North Carolina at Charlotte, 9201 University City Blvd., Charlotte, North Carolina 28223, USA

## Abstract

A contact-free diagnostic technique for examining position of the impurity energy level of p-type dopants in nitride semiconductors was proposed based on photoluminescence thermal quenching. The Mg ionization energy was extracted by the phenomenological rate-equation model we developed. The diagnostic technique and analysis model reported here are priorities for the design of highly effective p-doping of nitrides and could also be used to explain the abnormal and seldom analyzed low characteristic temperature *T*_0_ (about 100 K) of thermal quenching in p-type nitrides systems. An In-Mg co-doped GaN system is given as an example to prove the validity of our methods. Furthermore, a hole concentration as high as 1.94 × 10^18^ cm^−3^ was achieved through In-Mg co-doping, which is nearly one order of magnitude higher than typically obtained in our lab.

Advancements in p-doped nitrides, which were traditionally very difficult, often rely on optical based diagnostic techniques to examine dopant properties and position of the impurity energy level of the dopants^1^. As is well-known, when an impurity with less valence electron replaces the host atom in an otherwise perfect semiconductor, an acceptor impurity energy level is introduced. This impurity energy level is at an energy, *E*_*A*_, above the valence band maximum (VBM). In terms of Mg-doped nitrides systems, the impurity energy level corresponds to the 2*p* states of the Mg atoms^2^,^3^,^4^,^5^. *E*_*A*_ refers to the energy difference between impurity energy level and the VBM of the host. In general, *E*_*A*_is called acceptor ionization energy. It can be understood as the energy required to excite an electron from the VBM and leave a free hole in the valence band. Dopants with an impurity energy level close to the VBM will facilitate acceptor activation and display better p-type doping capabilities. Therefore, reliable diagnostic techniques for the determination of the energy position of impurity levels provide guidance for the design of highly effective p-doped nitrides.

In addition to the Hall measurement, of which the result is largely influenced by the ohmic contacts of the measurement pattern and the measurement conditions, the temperature dependence of the defect-related photoluminescence (PL) could been used to determine impurity energy level in nitrides (or to extract the ionization energy). The intensity of the PL emission typically decreases monotonically with increasing temperature, and is known as “thermal quenching”. As known, the underlying physical mechanism of thermal quenching is the redistribution of carriers into different energy levels (i.e. impurity level, valence band maximum, or conduction band minimum of the host material) following the excitation. These processes dominate the competition between different recombination channels. Therefore, PL intensity can be used to determine the characteristics of given point defects/impurities and carrier transitions in semiconductors^6^,^7^. Until now, most of the previous thermal quenching analysis has focused on n-type nitrides systems or undoped nitrides systems, and was used to extract a qualitative estimate of acceptor ionization energy^6^,^7^,^8^. Relatively few p-type nitrides systems have been studied because of the increased number of transitions and the more complicated band structure (usually acceptors and unintentional donors coexist)^7^, particularly in highly doped ones. However, these p-type systems are more important in nitrides.

In this work, temperature dependence of the defect-related PL intensity of In-Mg co-doped GaN samples was systematically analyzed. A phenomenological rate-equation model, considering both the thermal escape of both bound holes from acceptor levels and bound electrons from donor levels, was proposed and taken as a diagnostic technique to examine the Mg ionization energy. The validity of our methods was proved experimentally. Furthermore, our results also demonstrated that In-Mg co-doping could act as an attractive solution for achieving efficient p-type doping in high bandgap nitride semiconductors.

## Results

### Preparation of In-Mg co-doped GaN

Samples in this study were grown on 2 inch (0001) sapphire substrates using metal organic chemical vapor deposition (MOCVD). An 1 μm undoped GaN layer was deposited first on the substrate, followed by a 0.5 μm GaN:Mg layer at 1050 °C. The GaN:Mg layers were grown with trimethylindium (TMIn) at flow rates varying from 0 sccm to 135 sccm and a bis(cyclopentadienyl) magnesium (Cp_2_Mg) at a flow rate of 80 sccm. Thus, the TMIn/Cp_2_Mg molar flow ratios for our three samples were 0 (sample I), 30 (sample II), and 100 (sample III). To suppress the formation of compensation center (nitrogen vacancies), a high V/III molar flow ratio of 10000 was used. We used sample I as the reference. The evolution of the PL thermal quenching behavior was characterized using room-temperature photoluminescence (RTPL) excited with a 325 nm He–Cd laser. The laser power used was 20 mW with an estimated power density 2000W/cm^2^.

### Temperature dependent PL study

**Figure 1:**
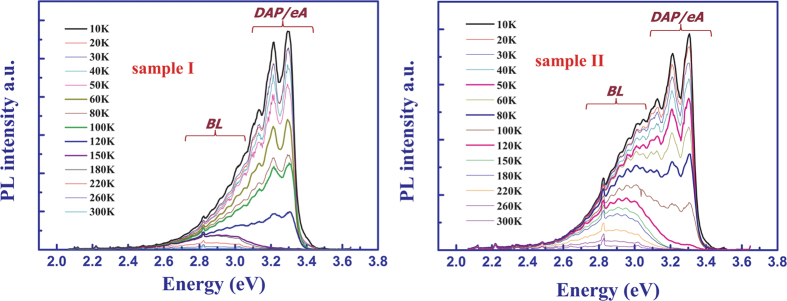
Evolution of PL spectrum in Mg doped (sample I) and In-Mg codoped (sample II) GaN with increasing temperature. Two sets of defect-related PL bands can be clearly resolved. One set is the ultraviolet luminescence (UVL) band (from 3.1 to 3.35 eV). Another set is the blue luminescence (BL) band (from 2.2 to 3.1 eV) peaking at 2.9 eV which should be attributed to V-N related deep donors. The characteristic temperature *T*_0_, at which the abrupt quenching begins, seem to decreases with increasing In concentration. Furthermore, the obviously enhanced blue luminescence indicates the increasing of hole concentration in sample II.

**Figure 2:**
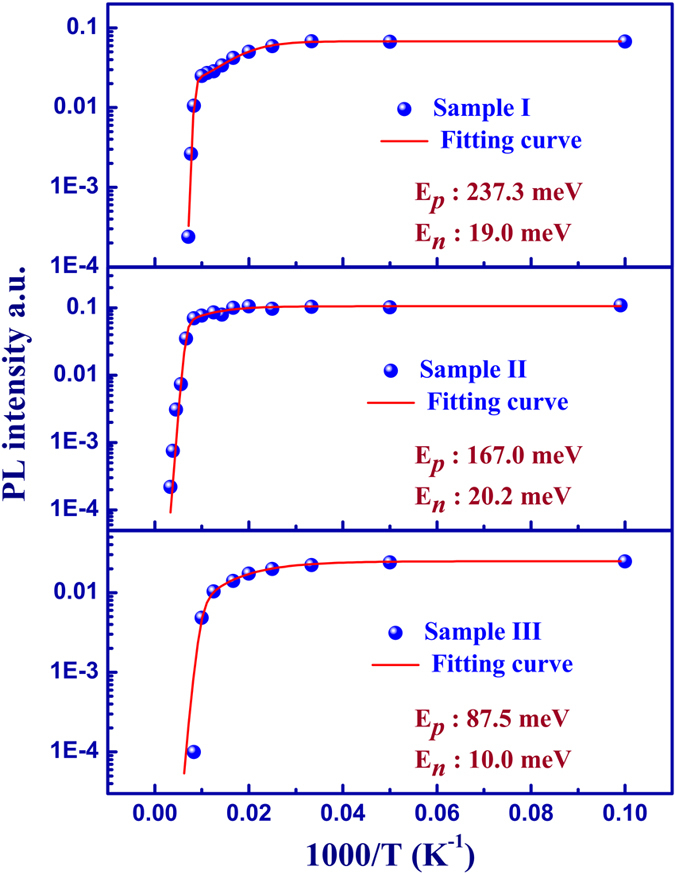
Temperature dependence of the PL intensity of *UVL*. Fitting curves are shown as solid lines. The ionization energy of acceptors and donors were also shown.

Figure 1 shows the evolution of the PL spectrum at selected temperatures ranging from 20 K to 300 K. For simplicity, only the spectrum of sample I and sample II are shown. Two sets of the defect-related PL bands can be clearly resolved by Gaussian fitting. One set is the ultraviolet luminescence (*UVL*) band (from 3.1 eV to 3.35 eV) with the main peak at 3.3 eV and its LO phonon replicas, originating from shallow donor-acceptor pair (*DAP*) and/or conduction-band-acceptor (*e-A*) transition^9^,^10^,^11^. At elevated temperatures, the *DAP*-type transitions are gradually replaced by the *e-A* transitions involving the same shallow acceptor. The shapes of the *DAP* and *e-A* bands are similar because they share the same acceptor level and have a relatively small donor ionization energy. As a result, they cannot always be discriminated from each other^11^,^12^,^13^,^14^. The other set is the blue luminescence (*BL*) band (from 2.2 eV to 3.1 eV) with the main peak at 2.9 eV, which is attributed to nitrogen vacancy (V_N_) related deep donors^15^,^16^,^17^. The *UVL* band dominates at low temperatures, and decreases slowly as the temperature increases from 10 K to about 80 K. The *UVL* band displays an abrupt quenching in the temperature range of 80 K to 180 K. At further increasing temperatures, the *UVL* band gives way to BL band and disappears under the tail of the BL band^9^,^18^. The PL intensity temperature dependence is shown in Fig. 2. The total decrease in the *UVL* band intensity is more than two orders of magnitude in the temperature range of 20 –300 K. It is worthy to note that the characteristic temperature *T*_0_, at which the abrupt quenching begins, decreases as In concentration increases. For instance, the *UVL* band from sample I can still be resolved at 100 K. However, the *UVL* band from sample II could not be detected.

### Diagnostic techniques for the determination of the energy position of impurity levels

As mentioned above, thermal quenching originates from the redistribution of carriers following excitation and is largely determined by the transition energy. From this point of view, thermal quenching can provide useful information regarding the nature of a given optical transition and the character of impurities, impurity ionization energy in particular. To get a better understand of the thermal quench behaviors in our p-type samples and analyze the different origin, it is useful to review the features of thermal quenching that are commonly observed in n-type GaN^6^. The Fermi level is very close to the conduction band minimum (CBM) in a n-type GaN. Therefore, it can be assumed that electrons are abundantly available for recombination. For this reason, the concentration of holes captured by the acceptor determines the temperature dependence of the *UVL* emission. As temperature increases, holes ionize to the valence band and result in the quenching of *UVL* transition. In other words, the defect-related transition is dominated by the concentration of the minority in n-type GaN^6^. In contrast, for Mg-doped p-type GaN, the concentration of holes is more sensitive to temperature due to the high acceptor ionization energy. Therefore, the quenching behavior should be quite different compared to n-type materials. One possible mechanism of thermal quenching for the p-type system is the thermal releasing of bound holes from the Mg acceptors to the valence band at elevated temperatures,similar as the circumstance of n-type. However, the ambiguity comes from the surprisingly low characteristic temperature *T*_0_ (about 100 K) of the thermal quench, where only a negligible fraction of bound holes can be ionized to the valence band due to the high ionization energy. Therefore, other mechanisms must coexist to fully encompass the thermal quenching. Another possible mechanism is that photo-generated electrons captured by donor levels return back to the conduction band at elevated temperatures. The release of electrons from donor levels to conduction band, on the one hand, may enhance e-A, but also enhance the capture to N_s_, which results in nonradiative recombination and the thermal quenching. Considering the relatively small donor ionization energy, the nonradiative combination processes may be enhanced even at cryogenic temperatures. This is consistent with our experimental observation. Therefore, considering the relatively low characteristic temperature, we argue that both the ionization process of acceptors and the ionization process of donor should be considered simultaneously for the analysis of PL thermal quenching in p-type GaN systems. Since e-A and DAP transitions cannot be easily resolved in the whole temperature range, and both transitions involve the same acceptor state, we analyze the thermal quenching of the UVL band as a whole to extract the acceptor binding energy.

Here, a phenomenological rate-equation model, considering both the thermal escape of both bound holes from acceptor levels and bound electrons from donor levels, was proposed and taken as a diagnostic technique to examine the Mg ionization energy. It is clear from Eq. (7) of Methods that the temperature dependence of PL intensity is largely determined by the process of the thermal ionization of acceptors into the valence band and the process of the thermal ionization of electrons into the conduction band, simultaneously. As shown in Fig. 2, the values of *E*_*p*_ and *E*_*n*_ can be obtained by iteratively fitting the PL intensity as a function of temperature. The acceptor ionization energy value, *E*_*p*_, decreases gradually from 237.3 meV in the reference sample I to 87.5 meV in sample III as In atoms were introduced. Such an obvious reduction to the acceptor ionization energy indicated that In incorporation could be used to achieve effective p-type doping in nitrides. The underlying physic of such In-Mg co-doping systems will be discussed in the latter part of this work.

### Temperature dependence hall measurement

**Figure 3:**
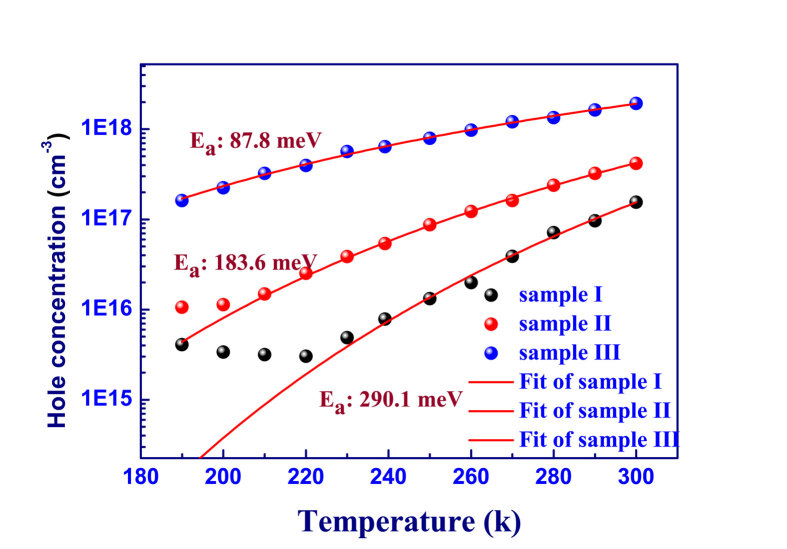
Hole concentration as a function of temperature. The fitting curves are shown as solid lines

Temperature dependence hall measurement was performed to further test the results of our PL thermal quenching analysis, as shown in Fig. 3. The measured hole concentration for reference sample I was approximately 1.55 × 10^17^ cm^−3^. As In incorporation increased, the hole concentration for sample II approached 4.2 × 10^17^cm^−3^. Further increasing the In concentration resulted in the increase of hole concentration to approximately 1.94 × 10^18^ cm^−3^. The acceptor activation was determined by fitting the concentration data, as shown in Fig. 3, and was consistent with the results from the thermal quenching PL quenching analysis. These results can be taken as a validation of our proposed methods.

## Discussion

Isoelectronic doping with In has shown potential for the enhancement of nitrides hole concentration^19^,^20^,^21^,^22^. The underlying physic could be understood from either the perspective of suppressing compensation^21^,^22^,^23^,^24^, or band coupling that formed a “new” higher VBM^4^. It is believed that it is critical to suppress the formation of N vacancy to achieve high efficient in such co-doping system due to the weaker strength of In-N bond than Ga-N bond^23^. In this work, extremely high V/III ratios (10000:1) and high growth temperatures (1050 °C) are used to yield sufficient amounts of active nitrogen species for gallium nitride growth, which is helpful for ammonias cracking and transport of atomic N to proper lattice sites. The strain analyses by XRD published by our group previously have confirmed the elimination of nitrogen vacancies in Indium doped nitrides by using non-equilibrium growth techniques^24^, which is consist with the results of Chung^25^. To get more effective p doping for nitrides, better approaches to suppress N vacancy are still highly desired.

In summary, a diagnostic technique using thermal quenching analysis was proposed to examine the energy position of impurity energy level of the dopants, which is helpful for the design of highly effective p-doping. A phenomenological rate-equation model, considering both the thermal escape of bound holes from acceptor levels and the thermal escape of bound electrons from donor levels, was develop to exam the Mg ionization energy. Compared to conventional Hall measurements, the proposed method is contact-free and therefore not influenced by the ohmic contact condition. The model reported here can also be used to explain the abnormal and seldom-analyzed low characteristic temperature, *T*_0_ (about 100 K) of thermal quenching in p-type nitrides systems. Finally, we demonstrated a hole concentration as high as 1.94 × 10^18^ cm^−3^, which is nearly one order of magnitude higher than typical value in our lab, could been achieved. These results can be taken as a validation of our proposed methods.

## Methods

### Phenomenological rate-equation

To test the validity of our assumption and quantitatively describe the temperature dependence, the different thermal quenching behaviors in our three p-type samples were analyzed based on the above considerations.

**Figure 4:**
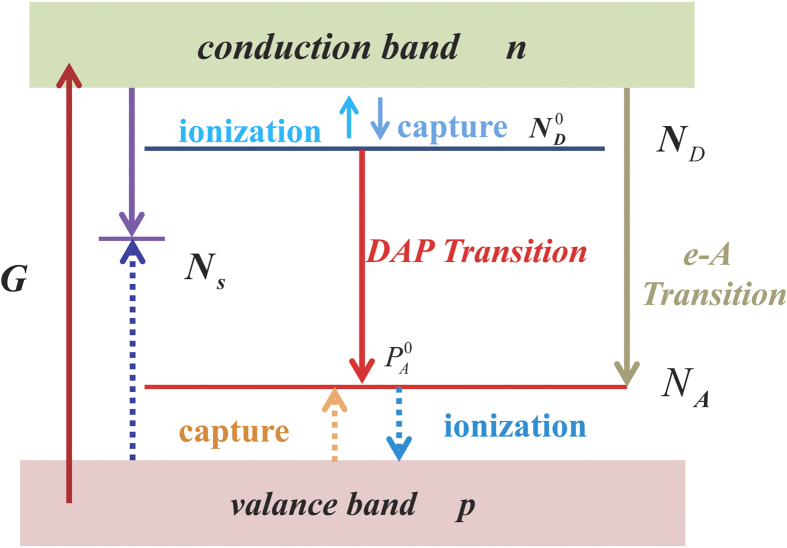
Band diagram and main transitions for a p type semiconductor. Here we consider a p type semiconductor containing three types of point defects, shallow donor, shallow acceptor, deep nonradiative center with concentration N_D_, N_A_, N_s_. Transitions of electrons and holes are shown with solid and dashed arrows, respectively.

First, let us consider the electron transition as shown in Fig. 4. Assume N_D_ and N_A_ are the n-type and p-type doping concentration, respectively. The rate of the electrons in the conduction band captured by donors after optical excitation could be expressed as 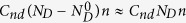, where 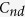
is the electrons capture coefficient for the donor, 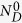 is the concentration of electrons at donor levels (neutral donors), and n is the conduction band (CB) electron concentration. In a p-type material at low excitation intensity, 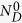
<<
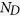
11. Another competing process is the free-to-bound radiative recombination between CB and the acceptor level at a rate of 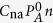, where *C*_na_ is the electrons capture coefficient for the acceptor, and 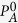 is the concentration of holes at acceptor levels (neutral or non-ionized acceptors). The exciton peak cannot be seen in our PL spectrum even at low temperature. Therefore, it is safe to neglect the contribution of the excitonic transition. Inevitably, some electrons will recombine through deep impurity or defect centers. The nonradiative recombination rate can be expressed as *C*_*ns*_*N*__s__*n*, where *C*_*ns*_ is the electron capture coefficient for the deep center, and N_s_ is the concentration of the deep centers. At elevated temperature, the bound electrons at donors may return to the conduction band as the result of thermal activation. The probability of this process is proportional to 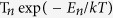, where *E*_*n*_ is the thermal activation energy for the donors, T_n_ is a constant, and k and T are the Boltzmann constant and temperature, respectively. In term of electrons captured by the nonradiatvie centers, it is assumed that they will recombine with a short lifetime and cannot return back to the conduction band at the temperatures used in our experiment. Similarly, the electrons at the donor levels will recombine radiatively at a rate of 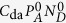.

Taking into account all the above processes, the kinetic equations under steady-state for the conduction band and the donor levels are given by (see the details in the Supplementary Information)


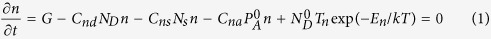



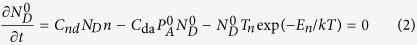


In Eq. (1), the first term is the inter-band generation rate. The second term describes the electrons captured by the donor levels. The third term is the loss of “non-radiative” through the deep states. Note that the recombination loss at the deep centers can be either radiative or nonradiative, including “BL”. We refer all these recombination channels as “non-radiative”, as far as the band edge radiative recombination is concern. The fourth term is the free-to-bound recombination, the fifth term the process of electron ionization back to CB.

The total UVL which can be calculated as


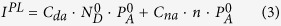


By solving Eqs (1–3), one can arrive at the results below:


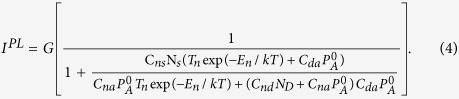


It is apparent that letting C_ns_ = 0, one would have I^PL^ = G, i.e., the PL efficiency would be 100%. Furthermore, if letting T_n_ = 0 (i.e., the electrons will not be re-emitted to the CB), one would have


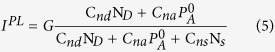


where the UVL efficiency is determined by the ratio of the two radiative electron depletion channels over the all three electron depletion channels. Therefore, in Eq. (4) the second term in the bracket represents the loss through the deep centers, which is determined by C_ns_ but thermally enhanced by the 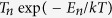 term at elevated temperatures.

Next, let us consider the hole transitions. At elevated temperatures, the bound holes at the acceptors may return to the valence band due to thermal activation, which is another primary mechanism, in addition to the electron thermal ionization of the donor centers, for the thermal quenching of UVL. At low excitation condition, in a p-type material, the hole concentration of the acceptor level can be approximated by the thermal distribution,


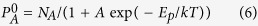


where A is a constant, and E_p_ is the acceptor binding energy. Substituting 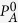 into Eq. (4), we have the equation below for the temperature dependence of the UVL:


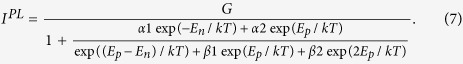


## Additional Information

**How to cite this article**: Liu, Z. *et al*. Analysis of Photoluminescence Thermal Quenching: Guidance for the Design of Highly Effective p-type Doping of Nitrides. *Sci. Rep.* **6**, 32033; doi: 10.1038/srep32033 (2016).

## Supplementary Material

Supplementary Information
