# Supplementary material for: Analysis of Photoluminescence Thermal Quenching: Guidance for the Design of Highly Effective p-type Doping of Nitrides
Source: Sci Rep. 2016 Aug 23;6:32033. doi: 10.1038/srep32033 (PMC4994085; doi:10.1038/srep32033)
Supplement: Supplementary Information [file srep32033-s1.docx]

**Supplementary Information**

***Analysis of Photoluminescence Thermal Quenching: Guidance for the Design of Highly Effective p-type Doping of Nitrides***

Zhiqiang Liu^1,2*^, Yang Huang^1,2^， Xiaoyan Yi^1,2^, Binglei Fu^1,2^, Guodong Yuan^1,2^, Junxi Wang^1,2^, Jinmin Li^1,2^, Yong Zhang^3*^

^1^Research and Development Center for Solid State Lighting, Institute of Semiconductors, Chinese Academy of Science，Beijing, 100086, China

^2^State Key Laboratory of Solid State Lighting, Beijing, 100086, China

^3^Department of Electrical and Computer Engineering, The University of North Carolina at Charlotte, 9201 University City Blvd., Charlotte, North Carolina 28223, U.S.A

**Rate-equation for thermal quenching**

In this study, it is assumed that the capture rates are much faster than recombination rates. This assumption is supported by experimental results[[1]](#endnote-1). Assume N_D_ and N_A_ are the n-type and p-type doping concentration, respectively. The rate of the electrons in the conduction band captured by donors after optical excitation can be expressed as $C_{nd}(N_{D}-N_{D}^{0})n\approx C_{nd}N_{D}n$, where $C_{nd}$is the electrons capture coefficient for the donor, $N_{D}^{0}$is the concentration of electrons at donor levels (neutral donors), and n is the conduction band (CB) electron concentration. In a p-type material at low excitation intensity, $N_{D}^{0}$<<$N_{D}$[[2]](#endnote-2). Another competing process is the free-to-bound radiative recombination between CB and the acceptor level at a rate of$C_{\text{na}}P_{_{A}}^{0}n$, where$C_{\text{na}}$ is the electrons capture coefficient for the acceptor, and $P_{_{A}}^{0}$ is the concentration of holes at acceptor levels (neutral or non-ionized acceptors). The exciton peak cannot be seen in our PL spectrum even at low temperature. Therefore, it is safe to neglect the contribution of the excitonic transition. Inevitably, some electrons will recombine through deep impurity or defect centers. The nonradiative recombination rate can be expressed as $C_{ns}N_{s}n$, where $C_{ns}$is the electron capture coefficient for the deep center, and N_s_ is the concentration of the deep centers. At an elevated temperature, the bound electrons at donors may return to the conduction band as the result of thermal activation. The probability of this process is proportional to T_n_$\exp(-E_{n}/kT)$, where $E_{n}$ is the thermal activation energy for the donors, T_n_ is a constant, and k and T are the Boltzmann constant and temperature, respectively. In terms of electrons captured by the nonradiatvie centers, it is assumed that they will recombine with a short lifetime and cannot return back to the conduction band at the temperatures used in our experiment. Similarly, the electrons at the donor levels will recombine radiatively with holes in the acceptor levels at a rate of $C_{\text{da}}P_{A}^{0}N_{D}^{0}$.

Taking into account all the above mentioned processes, the kinetic equations under steady-state for the conduction band and the donor levels are given by

$\frac{\partial n}{\partial t}=G-C_{nd}N_{D}n-C_{ns}N_{s}n-C_{na}P_{_{A}}^{0}n+N_{D}^{0}T_{n}\exp(-E_{n}/kT)=0$ , (1)

$\frac{\partial N_{D}^{0}}{\partial t}=C_{nd}N_{D}n-C_{\text{da}}P_{_{A}}^{0}N_{D}^{0}-N_{D}^{0}T_{n}\exp(-E_{n}/kT)=0$. (2)

In Eq. (1), the first term is the inter-band generation rate. The second term describes the electrons captured by the donor levels. The third term is the loss of “non-radiative” through the deep states. Note that the recombination loss at the deep centers can be either radiative or nonradiative, including “BL”. We refer all these recombination channels as “non-radiative”, as far as the band edge radiative recombination is concern. The fourth term is the free-to-bound recombination, the fifth term the process of electron ionization back to CB.

By solving Eq. (1) and Eq. (2), one can arrive at the results below:

$n=\frac{G\left(C_{da}P_{A}^{0}+T_{n}\exp(-E_{n}/kT)\right)}{C_{\text{da}}P_{A}^{0}(C_{nd}N_{D}+C_{ns}N_{s}+C_{\text{na}}P_{A}^{0})+(C_{ns}N_{s}+C_{\text{na}}P_{A}^{0})T_{n}\exp(-E_{n}/kT)}$ (3)

$N_{D}^{0}=\frac{GC_{nd}N_{D}}{C_{\text{da}}P_{A}^{0}(C_{nd}N_{D}+C_{ns}N_{s}+C_{\text{na}}P_{A}^{0})+(C_{ns}N_{s}+C_{\text{na}}P_{A}^{0})T_{n}\exp(-E_{n}/kT)}$, (4)

Since we only care about the total UVL which can be calculated as $I^{PL}=C_{da}\cdot N_{D}^{0}\cdot P_{A}^{0}+C_{na}\cdot n\cdot P_{A}^{0}$, (5)

$\begin{matrix} I^{PL}=G\left[\frac{C_{na}P_{A}^{0}T_{n}\exp(-E_{n}/kT)+(\mathrm{C}_{nd}N_{D}+C_{na}P_{A}^{0})C_{da}P_{A}^{0}}{C_{na}P_{A}^{0}T_{n}\exp(-E_{n}/kT)+(\mathrm{C}_{nd}N_{D}+C_{na}P_{A}^{0})C_{da}P_{A}^{0}+\mathrm{C}_{ns}\mathrm{N}_{s}(T_{n}\exp(-E_{n}/kT)+C_{da}P_{A}^{0})}\right] \\ =G\left[\frac{1}{1+\frac{\mathrm{C}_{ns}\mathrm{N}_{s}(T_{n}\exp(-E_{n}/kT)+C_{da}P_{A}^{0}}{C_{na}P_{A}^{0}T_{n}\exp(-E_{n}/kT)+(\mathrm{C}_{nd}N_{D}+C_{na}P_{A}^{0})C_{da}P_{A}^{0}})}\right] \end{matrix}$. (6)

It is apparent that letting C_ns_ = 0, one would have I^PL^ = G, i.e., the PL efficiency would be 100%. Furthermore, if letting T_n_ = 0 (i.e., the electrons will not be re-emitted to the CB), one would have

$I^{PL}=G\frac{\mathrm{C}_{nd}\mathrm{N}_{D}+C_{na}P_{A}^{0}}{\mathrm{C}_{nd}\mathrm{N}_{D}+C_{na}P_{A}^{0}+\mathrm{C}_{ns}\mathrm{N}_{s}}$, (7)

where the UVL efficiency is determined by the ratio of the two radiative electron depletion channels over the all three electron depletion channels. Therefore, in Eq.(6) the second term in the bracket represents the loss through the deep centers, which is determined by C_ns_ but thermally enhanced by the $T_{n}\exp(-E_{n}/kT)$ term at elevated temperatures.

Next, let us consider the hole transitions. At elevated temperatures, the bound holes at the acceptors may return to the valence band due to thermal activation, which is another primary mechanism, in addition to the electron thermal ionization of the donor centers, for the thermal quenching of UVL. The probability of this process occurring is proportional to$T_{p}\exp(-E_{p}/kT)$, where $E_{p}$ is the thermal activation energy for the acceptors, T_p_ is a constant related to the density of states in the valence band[[3]](#endnote-3). At low excitation condition, in a p-type material, the hole concentration of the acceptor level can be approximated by the thermal distribution,

$P_{A}^{0}=N_{A}/(1+A\exp(-E_{p}/kT)$, (8)

where A is a constant, and E_p_ is the acceptor binding energy. Substituting $P_{A}^{0}$ into Eq. (6), we have the equation below for the temperature dependence of the UVL:

$I^{PL}=\frac{G}{1+\frac{\alpha1\exp(-E_{n}/kT)+\alpha2\exp(E_{p}/kT)}{\exp((\mathrm{E}_{p}-E_{n})/kT)+\beta1\exp(E_{p}/kT)+\beta2\exp(2E_{p}/kT)}}$. (9)

1. Reshchikov, M. A. & Korotkov, R. Y. Analysis of the temperature and excitation intensity dependencies of photoluminescence in undoped GaN films. *Physical Review B* 64, 115205 (2001). [↑](#endnote-ref-1)
2. Reshchikov, M. A. & Morkoc, H. Luminescence properties of defects in GaN. *J. Appl. Phys.* 97, 061301 (2005). [↑](#endnote-ref-2)
3. Reshchikov, M. A. Two-step thermal quenching of photoluminescence in Zn-doped GaN. *Physical Review B* 85, 245203 (2012). [↑](#endnote-ref-3)
